# Kinematic parameters obtained with the ArmeoSpring for upper-limb assessment after stroke: a reliability and learning effect study for guiding parameter use

**DOI:** 10.1186/s12984-020-00759-2

**Published:** 2020-09-29

**Authors:** Nabila Brihmat, Isabelle Loubinoux, Evelyne Castel-Lacanal, Philippe Marque, David Gasq

**Affiliations:** 1 ToNIC, Toulouse NeuroImaging Center, Université de Toulouse, Inserm, UPS, Toulouse, France; 2 Department of Physical and Rehabilitation Medicine, University Hospital of Toulouse, Toulouse, France; 3 Department of Physiological Explorations, University Hospital of Toulouse, Toulouse, France; 4 Service des Explorations Fonctionnelles Physiologiques, Hôpital Rangueil, 1 Avenue du Pr Poulhes, 31059 Toulouse, France

**Keywords:** Learning, Hemiplegia, Exoskeleton device, Psychometrics, ArmeoSpring

## Abstract

**Background:**

After stroke, kinematic measures obtained with non-robotic and robotic devices are highly recommended to precisely quantify the sensorimotor impairments of the upper-extremity and select the most relevant therapeutic strategies. Although the ArmeoSpring exoskeleton has demonstrated its effectiveness in stroke motor rehabilitation, its interest as an assessment tool has not been sufficiently documented. The aim of this study was to investigate the psychometric properties of selected kinematic parameters obtained with the ArmeoSpring in post-stroke patients.

**Methods:**

This study involved 30 post-stroke patients (mean age = 54.5 ± 16.4 years; time post-stroke = 14.7 ± 26.7 weeks; Upper-Extremity Fugl-Meyer Score (UE-FMS) = 40.7 ± 14.5/66) who participated in 3 assessment sessions, each consisting of 10 repetitions of the ‘horizontal catch’ exercise. Five kinematic parameters (*task* and *movement time, hand path ratio, peak velocity, number of peak velocity) and* a global *Score* were computed from raw ArmeoSpring’ data. Learning effect and retention were analyzed using a 2-way repeated-measures ANOVA, and reliability was investigated using the intra-class correlation coefficient (ICC) and minimal detectable change (MDC).

**Results:**

We observed significant inter- and intra-session learning effects for most parameters except peak velocity. The measures performed in sessions 2 and 3 were significantly different from those of session 1. No additional significant difference was observed after the first 6 trials of each session and successful retention was also highlighted for all the parameters. Relative reliability was moderate to excellent for all the parameters, and MDC values expressed in percentage ranged from 42.6 to 102.8%.

**Conclusions:**

After a familiarization session, the ArmeoSpring can be used to reliably and sensitively assess motor impairment and intervention effects on motor learning processes after a stroke.

*Trial registration* The study was approved by the local hospital ethics committee in September 2016 and was registered under number 05-0916.

## Background

More than 40% of post-stroke patients display residual and permanent neurological upper extremity (UE) impairments [1]. It is essential to quantify these impairments in order to assess functional loss and develop more effective therapeutic interventions.

The effectiveness of motor rehabilitation is traditionally appraised using validated and standardized clinical scales [2], such as the upper extremity Fugl-Meyer subscale (UE-FMS) [3]. However, clinical scales are not always appropriate to assess motor strategies during movements, and they are not sensitive enough to capture the quality of sensorimotor performance or the effectiveness of therapeutic interventions [4]. They do not effectively distinguish between restitution and compensation [5, 6]. Some authors therefore recommend using kinematic parameters provided by optokinetic, robotic or gravity-supporting devices to assess movements [5–10]. These parameters are thought to be more sensitive and provide more information on movement performance and quality in the context of health and disease, helping to fill the gap related to the use of clinical scales.

Many robotic and non-robotic devices have been developed for UE rehabilitation after neurological disorders such as stroke [11, 12], with the goal of increasing the intensity and control of therapies. The ArmeoSpring (developed by Hocoma, Inc) is a passive orthosis that assists the movements of patients’ joints, using a structure parallel to the mobilized UE. It also provides kinematic parameters that inform about movement speed, duration and trajectory [9, 13], and thus could be used to assess movement efficacy and smoothness [7, 14]. Based on clinical criteria for impairments and function, the effectiveness of the ArmeoSpring was demonstrated in the rehabilitation of patients with motor deficits related to cerebral palsy, multiple sclerosis and stroke [8, 15, 16].

Given the increasing use of such devices as assessment tools, it is imperative to obtain better knowledge of the psychometric properties of the parameters provided [17, 18]. Indeed, these parameters must be sensitive enough to detect subclinical changes, and the variations observed must reflect a decrease in the motor deficit and not be due to a learning effect of the task. Some studies have addressed these questions [19–22]. Up to now, only one study has investigated the reliability of kinematic parameters provided by the ArmeoSpring [13]. Rudhe et al. demonstrated fair to good reliability of the movement workspace obtained with the ArmeoSpring in healthy participants and in patients with spinal cord injury [13]. Using mostly robotic devices, some authors have shown no or little learning effect [19–21] and advocated a single practice session to shorten the learning process. Other authors have demonstrated the existence of learning processes during mechanized training with the ArmeoSpring in post-stroke patients [23], and in children with cerebral palsy [16]. These latter studies used the vertical catch exercise, with only one or very few kinematic parameters used to assess motor learning and performance with the ArmeoSpring. Furthermore, motor learning is a fundamental process in rehabilitation and recovery post-stroke [6]. An increasing number of authors have suggested the use of kinematic parameters obtained with robotics to also assess motor learning and control in the contexts of health and disease. However, besides skill acquisition, motor learning also implies persistence of the changes brought about (i.e. retention) [24]. It is essential to at least demonstrate that the skills acquired are still present and measurable at a later time point. The majority of studies did not, however, address this question appropriately [24].

There is no consensus on the kinematic parameters to be used for UE assessment and little is known about their ability to identify learning during the post-stroke recovery phase. As far as we know, no study has investigated the extent of learning and its successful retention, together with the reliability of the parameters provided by the ArmeoSpring during the performance of a 2D-horizontal catch assessment exercise after a stroke. Thus, our main objective was to assess the learning effect and the reliability of the repeated measures of selected parameters obtained with the ArmeoSpring in post-stroke patients during their routine clinical care.

## Methods

### Participants

Thirty hemiparetic post-stroke patients were consecutively recruited during the course of their routine care in the Neurorehabilitation department of the Toulouse University Hospital. The routine care is standardized in accordance with the most recent guidelines for adult stroke rehabilitation and recovery [25] and with the French health authority [26]. Given the preliminary nature of this study for stroke, the sample size seemed appropriate and consistent with other studies [13]. All the patients included were naïve to the use of the ArmeoSpring and gave their written consents in accordance with the Declaration of Helsinki. The study was approved by the local hospital ethics committee in September 2016 (n°05-0916).

The inclusion criteria were: (i) a first ischemic or hemorrhagic stroke as diagnosed by a CT scan or MRI that occurred (ii) more than 3 weeks ago, (iii) an UE-FMS score between 10 and 44/66, and (iv) the presence of at least 10° voluntary movement at the shoulder and elbow. The exclusion criteria were: (i) the presence of apraxia, severe unilateral spatial neglect, (ii) UE pain limiting movement, and (iii) lack of stability of the trunk while seated or sitting position not recommended.

### Study design

**Fig. 1:**
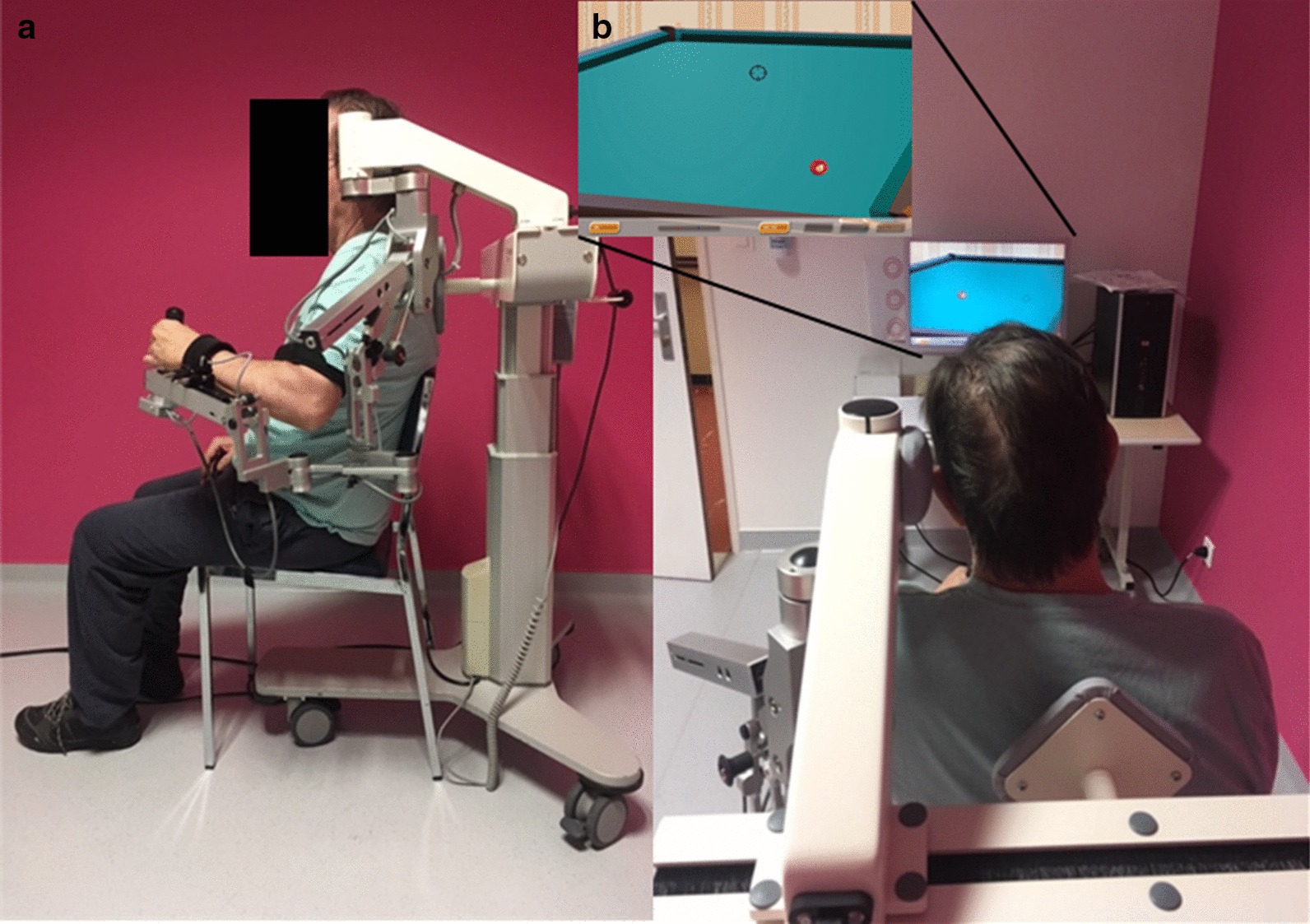
Experimental setup. **a** Installation of the patient performing a training exercise of the impaired upper limb with the ArmeoSpring. **b** Screenshot of the 2D-horizontal catch assessment exercise used in this study

Each patient made 4 visits over 2 days with the same unique rater who was an advanced user of the ArmeoSpring. During the pre-inclusion visit, the patients were informed by the rater about the protocol details, and the inclusion/exclusion criteria meeting was verified. If included, each patient made 3 visits on 3 consecutive half-days. During the first visit, the patient was comfortably seated on the ArmeoSpring, which was adjusted to allow movements of the UE in a large tridimensional workspace required to perform the assessment exercises (Fig. 1). During the second and third visits, the patient was placed on the device in the same way and performed the same series of exercises as during the first visit.

### The ArmeoSpring device

The ArmeoSpring (*Hocoma, Switzerland*), is a passive exoskeleton which provides UE weight support and allows early training of motor skills [27]. It has six degrees of freedom and can be attached to the UE at the level of the arm, forearm and wrist. It thus allows self-initiated arm movements in a large tridimensional workspace. Support against gravity is provided by adjustable springs for the upper arm (9 levels, from A, no tension exerted and minimum support, to I, maximum tension and support) and the forearm (5 levels from A to E). It is supplied with the *Armeocontrol 1.22* software, which provides many functional exercises, simulated in a virtual environment with auditory and visual feedback. The software also allows recording kinematic parameters via seven sensors positioned on the different exoskeleton joints, and provides all the exoskeleton joint angles, the effector location in a tridimensional workspace (used to control a cursor on a screen) and the grip pressure. Several assessment exercises are available, with different levels of difficulty. Difficulty can be modulated by the workspace size and the number of targets to be caught.

### Experimental procedures

All the patients were seated in the same standardized and ergonomic central position, in front of the computer screen (Fig. 1a). The exoskeleton was adjusted to the length of the arm and forearm, but the same level of weight support was set for all the patients (medium support, level E for the arm, and level C for the forearm). Initially, the patient's shoulder was placed between 0 and 20° elevation and elbow at 90° flexion. Patient-specific settings on the ArmeoSpring were retained between the three consecutive sessions.

Each testing session lasted between 20 and 30 min, depending on the patient’s motor impairment. Each patient performed with their paretic upper limb 10 repetitions (trials) of the same assessment exercise (the 2D-horizontal catch, Fig. 1b) separated by 30 s of rest. The 2D-horizontal pointing task required moving the cursor (corresponding to the patient’s hand) in order to catch the targets (represented by red billiard balls) that appeared sequentially on the screen. Depending on the position of the target in the workspace, the patients had to perform shoulder movements or a combination of shoulder and elbow movements in order to reach the most distant targets. Each patient was instructed to move as accurately as possible and at a self-selected speed, while being aware that they had a time limit of 10 s to catch each ball. When a ball was caught, it disappeared and another appeared at a new fixed location. During a trial, 12 balls had to be caught and the time to catch a ball was limited to 10 s; if this period was exceeded, the ball disappeared and another ball appeared at the new location. For this study, the difficulty of the 2D-horizontal catch exercise was set to the easiest level (level 1) for all the patients, with a predefined number of targets (12 targets) and a horizontal workspace size of 40 $\times$ 30 cm.

Each patient was subjected to three repetitions of the testing session, resulting in 30 trials per patient. The target positions and sequence order remained fixed throughout the 3 visits. The between-patient standardization of the protocol settings (compensation level, exercise difficulty and rest period) allowed attributing the potential differences between patients to performance changes rather than changes related to different settings. The sessions were controlled independently by the rater.

### Kinematic assessment

A unitary movement was defined between two consecutive targets and considered only if both balls, the previous and the next, were successfully caught. During a trial, a maximum of 12 balls had to be caught, thus representative of 12 consecutive unitary movements.

The Armeocontrol software records raw data, specific to the assessment exercise, at a frequency of 64 Hz, corresponding in this case to the hand position in the horizontal plane (XY), and the time when the target appeared, was caught and disappeared. From the raw data file, we computed kinematic parameters with a custom code implemented on Matlab software (see Additional file 1: S1)*,* freely downloadable at https://github.com/davidgasq/Armeo_2DHorizCatch.git. These parameters were chosen because, based on the recommendations by Schwarz et al. [5], they are relevant to explore different dimensions of the movement performed.

The *task time* (*TaskTime* in seconds, s) was the duration needed to complete the exercise (the maximal duration was 120 s). The *movement time *(*MovementTime*, s) was the duration given to catch one ball (10 s maximum per ball) and reflected the efficiency of movement. The *peak velocity* (*PeakVel*, cm/s) was the maximal absolute velocity recorded during each movement. The *hand path ratio* (*HPR*, dimensionless) was the ratio between the real path in the horizontal plane and the shortest possible one (a value ranging between 1 and infinity) and reflected movement efficiency. The *number of velocity peaks* (*nPeak*) was the number of peaks, defined as the number of times the derivative of velocity changes sign from positive to negative, and which reflected the smoothness of the movement. The *Score* (%) corresponded to the game score, computed as the number of balls reached divided by the total number of balls that could be reached, and summarized the efficiency of the movement.

The Armeocontrol software systematically provided a summary report where 3 parameters among those described above were given: *HPR*, *TaskTime* and *Score*.

### Data analysis

The statistical analyses were performed using Statistica software *(StatSoft. Inc. Version 10).* The significance threshold of the p-value was set at 0.05. For each trial and each patient, the parameter data were averaged from all the successful unitary movements (a maximum of 12 balls). The data were also averaged for each session (10 consecutives trials).

We first ensured that the kinematic parameters of the summary report and those calculated with the custom code were consistent (paired t-tests not statistically significant, see Additional file 1: Figure S2). Although we tried to standardize the starting position at the beginning of the exercise, we observed that not all the patients started from the same position. Some patients had their hands already almost placed over the ball. Accordingly, the first trajectory (corresponding to the movement which starts from the 1st ball caught) was excluded from the analysis. The number of failed attempts was also significantly decreased for the target 1, which supports our observation (see Additional file 1: Figure S3). Consequently, only the last 11 unitary movements were considered to compute the parameters. We detected outliers using the Tukey method [28] and removed them from the statistical analysis.

Secondly, the learning effect was studied using a 2-way repeated measures ANOVA (rm-ANOVA, 10 trials * 3 sessions) to determine if differences existed between the ten trials of each of the three sessions. The dependent variables were tested for non-sphericity using Mauchly’s test and those not meeting the sphericity assumption were adjusted using the Greenhouse–Geisser correction and corrected p-values were reported instead. If significant, a Tukey post-hoc analysis was applied to analyze significant main effects and interactions. If no trial * session interaction was found, we considered the same trial effect across sessions. The retention of the kinematic parameters was inferred from the rm-ANOVA results with the between-session comparisons. Indeed, the data obtained from the last day of training (S3) were compared to those obtained at the end of the previous day, during S2.

Thirdly, reliability was studied specifically on the averaged data of the sessions and trials for which we considered there was no longer an obvious learning effect (the last four trials of S2 and S3, see “Results” section for details). The relative reliability was evaluated using the intraclass correlation coefficients (ICC) that provide information on inter- and intra-session reliability. We used the ICC_2,k_ because we analyzed averaged data which were independent from the rater [29]. An ICC ≥ 0.75 was considered excellent, moderate if between 0.40 and 0.75 and weak if < 0.40 [30].

The MDC_95_ (minimal detectable change) represents the magnitude of change necessary to exceed the measurement error of 2 repeated measures at a confidence interval of 95% (CI_95%_) [31]. It integrates the variability of the measurement related to the patient, the tool and also possible systematic biases between test–retest sessions, such as a learning effect. A low MDC corresponds to a better theoretical capability of the parameter to detect a real change. First, the standard error measurement (SEM) was computed, considering the systematic differences between the test and retest, with the following formula:

$$SEM = \sqrt {\sigma \left( {intra} \right)^{2} + \sigma \left( {residual} \right)^{2}}$$

where $\sigma {\left(intra\right)}^{2}$ represented the variance of individual differences between the test–retest measurements and (*residual*)^2^; the residual variance of the interaction between intra- and inter-individual differences obtained from a repeated ANOVA [31]. Then, the MDC_95_ was computed as follows [32]:

$$MDC_{95}=1.96 \times SEM \times \sqrt{2}$$

MDC_95_ was also expressed as a percentage (MDC_%_) so that it could be independent of the measurement unit and comparable across the kinematic parameters, thus:

$$MDC_{\%} = \frac{MDC_{95}}{mean}\times 100$$

where the mean is the parameter averaged for all the observations across the selected trials of two sessions. Finally, the CI_95_ of the mean difference was computed between the test and retest measures to identify any systematic trends or outliers, and no residual systematic bias was considered if it included the zero [33].

## Results

All the 30 patients performed the 3 assessment sessions under the rater’s control. Only one patient (#27) performed 6 trials instead of 10 in each of the 3 sessions, due to fatigue. The mean age was 54.5 ± 16.4 years; the post-stroke time was 14.7 ± 26.7 weeks. The average UE-FMS was 40.7 ± 14.5 [from 15 to 65]. The detailed data for each patient are presented in Table 1.

**Table 1:** Patient characteristics

Patients	Gender	Age (years)	Dominant hand	Post-stroke time (weeks)	Paretic side	UE-FMS (/66)
1	M	74	R	4	L	29
2	M	69	R	3	L	30
3	M	54	R	22	L	24
4	F	76	R	8	L	42
5	M	49	R	16	L	48
6	M	23	L	18	R	57
7	F	70	R	10	L	64
8	M	59	R	6	R	15
9	M	61	R	5	L	39
10	M	62	R	8	L	38
11	F	34	R	11	R	15
12	M	48	R	7	R	33
13	F	76	R	20	L	36
14	F	72	R	7	R	51
15	F	33	R	9	R	22
16	M	46	R	3	R	54
17	M	76	R	14	R	36
18	M	53	R	7	R	28
19	F	47	R	10	L	31
20	M	65	R	153	L	28
21	M	35	R	3	R	48
22	F	38	R	10	R	48
23	M	33	R	11	R	65
24	M	69	L	15	L	53
25	M	67	L	4	L	42
26	M	43	R	5	L	44
27	M	52	R	20	L	23
28	M	59	R	5	L	55
29	F	70	R	8	R	58
30	M	22	R	66	R	64

*F* female, *L* left, *M* male, *R* right, *UE-FMS* upper extremity Fugl Meyer scale

### Learning effect

**Fig. 2:**
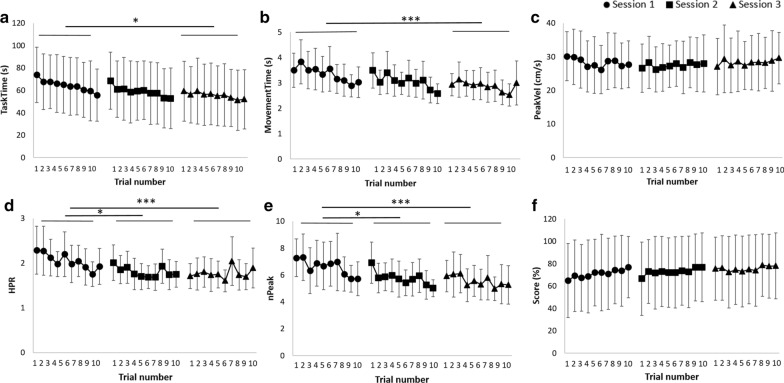
Learning curves of the averaged parameters (± SD) showing the evolution of a specific parameter over the trials (1 to 10) for the 3 sessions. **a** Task time (TaskTime, s). **b** Movement time (MovementTime, s). **c** Peak velocity (PeakVel, cm/s). **d** Hand path ratio (HPR, dimensionless). **e** Number of peak velocity (nPeak). **f** Game score (Score, %). Between-session significances are represented with asterisks (*p < 0.05; **p < 0.01; ***p < 0.001) and within-session significances are reported in Table 2

Most of the parameters showed an intra- and/or inter-session learning effect, independent from each patient’s initial performance (results not shown), corresponding to a significant improvement of the parameters across trials and/or sessions, respectively. The ANOVA values and the significant differences between sessions and/or trials are reported in Table 2. The learning curves are shown in Fig. 2.

**Table 2:** Learning effect analysis with a two-way repeated measures ANOVA

	Inter-session effect (among 3 sessions)		Intra-session effect (among 10 trials)										
	S1	F-value; p-value	F-value; p-value	T1	T2	T3	T4	T5	T6	T7	T8	T9	T10
TaskTime (s)	S3	4.23; p < 0.05	7.59; p < 0.0001	T2–T10	–	T9	–	–	–	–	–	–	–
MovementTime(s)	S3	9.71; p < 0.001	9.96; p < 0.0001	T7–T10	T7–T10	T9–T10	T9–T10	T9–T10	T9–T10	–	–	–	–
PeakVel (cm/s)	–	–	–	–	–	–	–	–	–	–	–	–	–
HPR	S2/S3	10.69; p < 0.001	5.74; p < 0.0001	T9–T10	T6, T9–T10	T9	–	T9	–	–	–	–	–
nPeak	S2/S3	11.16; p < 0.001	10.76; p < 0.0001	T4, T6–T10	T3–T4, T6–T10	T10	–	–	–	–	–	–	–
Score (%)	–	–	3.59; p < 0.01	T2, T5–T10	–	–	–	–	–	–	–	–	–

HPR, hand path ratio (dimensionless); MovementTime, movement time; nPeak, number of velocity peaks; PeakVel, peak velocity; Score, the game score corresponding to the number of balls reached divided by the total number of balls that could be reached; S1, session 1; S3, session 3; TaskTime, task time; T1 to T10, trials 1 to 10. The second column report the session(s) significantly different from session 1 (S1). The columns of the intra-session effect report the trial(s) significantly different from each other

A session effect was observed for most parameters, except for *PeakVel* and *Score *(Table 2). Tuckey post-hoc tests revealed that the session effect occurred mainly between S3 and S1 for *TaskTime* (p = 0.02) and *MovementTime* (p = 0.0006) and between S2/S3 and S1 for *HPR* (p = 0.026 and p = 0.0005 respectively) and *nPeak* (p = 0.037 and p = 0.0004 respectively). A trial effect was also observed for *nPeak* (p < 0.05, Table 2) and the learning effect was no longer observed after the 6th trial (four last columns of the Table 2). For *Score*, only a trial effect was highlighted between the first trial and the second on one part, and the 5th to the 10th on the other part (p < 0.05). No session or trial effect was shown for *PeakVel.*

Learning occurred mainly between S1 and S2/S3 (Fig. 2), which made us consider retention between S2 and S3. Indeed, no significant difference (p > 0.05, Table 2, Fig. 2) was observed between the kinematic parameters measured in S3 and those measured at the end of the previous day, in S2, thus revealing the successful retention of the skills acquired for all the kinematic parameters.

Additionally, the Additional file 2: Figure S4 represents the individual learning curves obtained from two patients’ data (patient #21 and #29). These patients had mild residual motor deficits (as reflected by their UE-FMS > 47/66 [34], Table 1). Their performance in the task are similar when looking at the *TaskTime* or *MovementTime* (Additional file 2: Fig. S4.A and B) and *Score* (reaching a score of 100/100, Additional file 2: Fig. S4.F) but not so much when looking at the *PeakVel*, *HPR* and *nPeak* parameters (Additional file 2: Fig. S4.C–E respectively).

### Reliability

Considering the previous ANOVA results and the graphical observation of the evolution of reliability (see Additional file 2: Figure S5 for details), data from trials 7 to 10 (the last 4 trials) of sessions 2 and 3 were selected for the reliability analysis. The reliability data are reported in Table 3. All the parameters, except *MovementTime*, *HPR* and *nPeak*, had excellent relative reliability, as expressed by the CI_95_ lower bound of the ICC ≥ 0.75.

**Table 3:** Reliability data for the 30 patients computed from the last 4 trials of sessions 2 and 3

	Mean S2 (SD)	Mean S3 (SD)	ICC (CI_95%_)	MDC_95_	MDC_%_	mDiff (CI_95%_)
TaskTime (s)	58.95 (31.73)	53.82 (31.16)	0.97 (0.91 to 0.99)	57.94	102.77	− 5.13 (− 9.13 to − 1.13)
MovementTime (s)	3.20 (0.74)	2.97 (0.77)	0.77 (0.47 to 0.90)	2.58	83.78	− 0.24 (− 0.50 to 0.03)
PeakVel (cm/s)	28.32 (9.97)	27.61 (9.29)	0.93 (0.83 to 0.97)	12.06	43.11	− 0.71 (− 2.85 to 1.43)
HPR	1.91 (0.39)	1.79 (0.36)	0.77 (0.47 to 0.90)	1.28	68.92	− 0.12 (− 0.25 to 0.02)
nPeak	6.25 (1.58)	5.61 (1.34)	0.78 (0.45 to 0.91)	6.51	109.77	− 0.64 (− 1.12 to − 0.16)
Score (%)	70.66 (36.81)	73.46 (36.90)	0.99 (0.97 to 0.99)	32.84	45.57	2.80 (− 0.003 to 5.59)

ICC, intraclass correlation coefficient (lower and upper-bound of the 95% confidence interval [CI_95%_]); MDC_95_, minimal detectable change values computed with a 95% confidence interval; MDC_%_, minimal detectable change values expressed as a percentage of the mean; mDiff, mean difference computed between test and retest measures (lower and upper-bound of the CI_95%_); Mean S2/S3, mean (standard deviation [SD]) value of parameters for session 2 (S2) and 3 (S3)

The MDC_%_ values were heterogeneous from one parameter to another, ranging from 43.1 to 109.8%, with only *PeakVel* and *Score* showing a MDC_%_ < 50%. For *TaskTime* and *nPeak,* a residual systematic bias (i.e. CI_95_ of *mDiff* not including zero) can be seen, reflecting an improvement between sessions 2 and 3.

## Discussion

In this pilot study, kinematic parameters computed from data provided by the ArmeoSpring exoskeleton were analyzed to investigate the relevance of these parameters in the assessment of post-stroke hemiplegic patients during a 2D-horizontal catching exercise. The results highlighted an intra- and inter-session learning effect for all the parameters except *PeakVel*. The reliability analysis, applied to data without a priori learning, showed that *PeakVel* and *Score* had the lowest margin of error.

### Learning effect

We observed an inter- and intra-session learning effect for the parameters *MovementTime, TaskTime*, *HPR* and *nPeak* and an intra-session effect only for the *Score*. This result highlights the importance of the learning effect, even most studies reported little or no learning effect for the kinematic parameters obtained with robotic and non-robotic devices [19–21, 35]. This difference may be explained by the fact that, unlike us, the authors of the latter studies used robotic devices. Such devices provide some assistance during movements, thus maybe limiting the learning process during the performance of the task. It has already been described that physical assistance hinders motor learning of a simple walking balance task in healthy subjects [36]. Furthermore, in order to shorten the learning process, the authors preconized a single practice session before the real training sessions, which may have also limited the learning effect observed during the latter*. MovementTime* was the parameter most sensitive to the learning effect, showing a significant decrease of the time needed to catch a ball across trials and sessions. This parameter is used to globally assess the patient's ability to perform the movement [7], reflecting movement efficiency [5], and is classified in the "activity" domain of the ICF [4]. Given the importance of this learning effect, it seems necessary to repeat the exercise at least ten times per session, and consider only the last four trials of the second session to obtain a consistent result. The entire first session and the first six trials of the following sessions should not be considered because *MovementTime* continues to decrease, independently of any intervention or recovery. *nPeak*, used to characterize the smoothness of the movement [4, 5] and which has been shown to decrease following robotic training [37], is also sensitive to a persistent inter- and intra-session learning effect. This learning effect was already described in post-stroke patients during a frontal plane reaching task with the ArmeoSpring [23]. In this latter study, the fast and early improvement of this parameter was considered to reflect the improvement of performance due to learning processes, while its late and slower improvement was considered to reflect a reduction of UE motor impairments. However, in view of the design of our study, which took place over only 2 days, we cannot extrapolate this latter result. The parameters that showed a persistent learning effect over sessions may be used to assess the effect of a specific therapeutic intervention on learning processes that are known to occur in post-stroke settings [38, 39].

The *PeakVel* and *Score* parameters were less sensitive to the learning effect. *PeakVel,* which evolves with time post-stroke to match healthy patient values [12], showed a concurrent validity with the UE-FMS score [9] and moderate quality of evidence regarding its reliability [5]. However, within a session, the *Score* stabilized quickly after 1 trial. It was shown to correlate with wrist function [40] and reflects movement efficacy [5]. Consequently, these two parameters should be used to assess patient performance/impairment and motor recovery at a given time or over time. Although considered similar, *MovementTime* and *PeakVel* showed different sensitivity to the learning effect. This may be due to the fact that these parameters reflect different aspects of movement properties. As already mentioned, *MovementTime* reflects a global dimension of the temporal efficiency of a movement [5, 17]. This parameter is correlated and predicts well the residual motor deficits of stroke patients as assessed with the UE-FMS [41], thus it is recommended for the evaluation of motor recovery and robot-assisted rehabilitation after stroke [4, 42]. *PeakVel* is a speed metric that reflects the first (i.e. ballistic) phase of a movement, its strategy and ease [17]. Contrary to movement duration, *PeakVel* showed weak correlation with clinical scales [21, 42] and less sensitivity to changes [37, 43]. These arguments may explain their different sensitivities to the learning effect. Whereas patients continued to perform the movements in an increasingly shorter time, *PeakVel* remain unchanged and the time of occurrence of the peak velocity during the movement increased across session (see Additional file 2: Figure S6), thus revealing a right-shift of the velocity profile. This result is in favor of the improvement of the corrective and controlled phase of the movement across sessions [44, 45]. Additional file 2: Figure S4 also highlights the importance of a kinematic assessment with different parameters from those computed by the Armeocontrol to highlight subtle differences between subjects, not shown by UE-FMS, or due to recovery and/or therapeutic intervention.

Retention refers to the persistence of the performance acquired during the training period. This phenomenon is an important part of motor learning [24]. The gains in all the kinematic parameters chosen were retained for at least 24 h (as revealed by the absence of significant differences between S2 and S3). These results revealed the successful inter-session retention of the 2D-horizontal exercise with this paradigm in our stroke population and are in line with previous studies [16].

### Reliability

All the parameters selected showed overall an excellent (*TaskTime, PeakVel* and *Score*) or moderate (*MovementTime*, *HPR* and *nPeak*) relative reliability [30]. These results are consistent with those of other studies investigating this type of task in stroke populations [19, 46, 47]. Thus, they may be appropriate for intra-individual comparisons [35].

MDC_95_ and MDC_%_ are useful in determining whether a change of a parameter is metrically real or if it is due to a measurement error. Thus, the lower the measurement error, the greater the reliability [47–49]. For a patient, a significant improvement may therefore be suggested when the improvement of the parameter exceeds the MDC_95_ values reported in Table 3. MDC_%_ values ranged from 42.6 to 109.8%, indicating that some parameters require larger variations than others to highlight real changes. For *TaskTime*, its variation must exceed 102% to indicate a real change, which is congruent with the literature [17, 19, 47, 50]. For example, in a study assessing stroke patients performing a simple forward-reaching task measured with an optical tracking system, MDC_%_ ranged from 7.4 to 98%, depending on the kinematic parameter, the task instructions and the analysis method used [47]. For the *HPR*, the MDC_%_ ranged between 7.4 and 28.9%, whereas the values ranged between 24.4 and 67.6% for the *nPeak* [47]. The higher values found in our study for these parameters could be explained by the method of MDC computation we used which, unlike [47], incorporated the presence of a systematic bias between tests and retests [31]. Although we have shown that there are still systematic bias residues (i.e. learning effect) when calculating the reliability between sessions 2 and 3, our MDC values are higher but may better reflect the reality of clinical practice.

### Important considerations and limitations

Since we wanted unrestricted arm movements, the exoskeleton was unlocked at the level of shoulder and elbow. Sometimes, the hand was directly located at the first ball position and therefore the movement observed did not reflect the real one. Consequently, in our study, the data were averaged over 11 consecutive movements and not 12 as designed in the horizontal catching task, and as the Armeocontrol software computes kinematic parameters.

We investigated the psychometric properties of certain carefully chosen kinematic parameters based on a recent review [5], that represent all the dimensions of a movement. The kinematic parameters were slightly different (although not significantly) from those provided in the ArmeoSpring report (see Additional file 1: Figure S2), but computed with a stricter and more rigorous methodology (removal of the first target, trajectories considered only if the departure and arrival targets are reached). This may be used to administer a short assessment protocol to post-stroke patients with the ArmeoSpring, but could also limit its ease of use in routine care by a clinician. To exceed these limits, we have made available to the community the script used to calculate the parameters (see Additional file 1: S1).

Depending on the research questions and hypothesis, some parameters may be more appropriate than others to capture movement patterns. As demonstrated, we must be careful in the interpretation since the initial parameter value may also depend on learning processes that are relatively independent from the impairment reductions [23]. In our study, learning occurred mainly between session 1 and sessions 2 and 3, and until the sixth repetition for some parameters. Consequently, in similar conditions and particularly for the parameters *MovementTime*, *TaskTime*, *HPR* and *nPeaks*, we suggest considering the first session (consisting of 10 repeated trials) as a session of familiarization with the device and the task to avoid data corruption by learning processes. For the learning effect to be minimized, the actual assessment session should include a minimum of 1 to 6 trials, depending on the parameter used (see Table 2). The measurement error data computed in our study are applicable for judging a change over time (e.g., pre-post treatment) only if 10 trials are performed and the last 4 averaged. However, some MDC values were still high and variable across parameters, with a systematic bias for some of them. It may therefore be more relevant to identify for each parameter a specific number of trials per session to overcome the learning effect observed.

We cannot exclude the influence of the exoskeleton support on the results since some devices are known to affect the validity of kinematic data [9, 51]. However, since the ArmeoSpring is a passive orthosis, we can assume that it was limited. A comparison with the kinematic parameters obtained during the same task but without weight support and with a free UE may appropriately address this question.

Unfortunately, we were not able to assess the successful transfer or generalization of the task, which is another important aspect of motor learning. A transfer test is usually administered after the training period and assesses the skill with another effector or a skill that was not practiced [24], thus revealing the effects of learning on untrained effectors/contexts/tasks. It would be interesting to carry out further studies to evaluate retention over a much longer period of time and generalization to other functionally relevant tasks [52, 53].

## Conclusions

This study demonstrated that the ArmeoSpring may be effectively used for a reliable, objective and quantitative assessment of upper-extremity motor and functional impairments, and to assess therapeutic effects on motor learning in post-stroke patients. The results provided greater precision for structuring an assessment session with the device, depending on the research question. An initial session with a specific number of trials (depending on the parameter) must be performed to allow the patient to familiarize themselves with the procedure, before carrying out the actual assessment sessions. Certain parameters such as *PeakVel* and *Score* may be used to assess performance at a specific time whereas *TaskTime, MovementTime*, *HPR* and *nPeak* may be used to assess the effect of specific interventions on learning processes. This preliminary study confirms the importance of such studies aimed at standardizing the use of kinematic assessment, and emphasizes the relevance of using such devices to track and highlight subtle changes and progress due to learning, recovery and the administration of therapeutic interventions.

## Supplementary information

**Additional file 1.** Additional methodological file.** Additional material S1. **Custom Matlab code. The Matlab code used for the calculation of kinematic parameters, available on GitHub website. **Figure S2.** Box plot comparison between kinematics calculation methods. Comparison between the averaged parameter values provided in the summary report of the ArmeoSpring (Armeocontrol Software) and those calculated with the Matlab code (custom code). Statistical results of the paired t-tests for the hand path ratio (A. HPR), Task Time (B. TaskTime in seconds) and the Score (C. Score in percentage) are reported on the figure. **Figure S3.** Graphical representation of the average number of failed attempts to catch the consecutive balls (targets 1 to 12). Statistical parameters of the ANOVA are shown below the graph. The number of failed attempts to catch target 1 is significantly lower compared to the other targets (*p < 0.0001).

**Additional file 2.** Additional results file.** Figure S4. **Learning curves of two example patients’ data (#21 and #29) showing the evolution of a specific parameter over the trials (1 to 10) for the 3 sessions. A. Task time (TaskTime, s). B. Movement time (MovementTime, s). C. Peak velocity (PeakVel, cm/s). D. Hand path ratio (HPR, dimensionless). E. Number of peak velocity (nPeak). F. Game score (Score, %). **Figure S5.** Evolutions of Minimal Detectable Change (MDC, dashed lines) and Intraclass Correlation Coefficient (ICC, solid lines) according to the number of trials (of sessions 2 and 3), taking into account the calculation of reliability (1: only the 10th trial, to 10: the 10th to the 1st trial). Different parameters are represented by different colors and shapes (black (■): peak velocity, gray (♦): score, orange (●): number of peaks, blue (▲): task time, green (⁃): movement time, yellow (▬): HPR). **Figure S6. **Learning curves of the averaged time of occurrence of the peak velocity during the movement expressed in percentage (PercPeakVel (%) ±SD), showing the evolution of this parameter over the trials (1 to 10) of the 3 sessions. Between-session significance is represented with asterisks (* p < 0.05). No significant trial effect was observed.

## Data Availability

The dataset used and analyzed during the current study is available upon reasonable request from the corresponding author.

## Abbreviations

UE: Upper extremity
UE-FMS: Upper extremity Fugl-Meyer subscale
TaskTime: Task time
MovementTime: Movement time
PeakVel: Peak velocity
HPR: Hand path ratio
nPeak: Number of velocity peaks
ICC: Intraclass correlation coefficient
MDC_95_: Minimal detectable change at a confidence interval of 95%
SEM: Standard error measurement
MDC_%_: Minimal detectable change expressed as a percentage
