# Supplementary material for: Kinematic parameters obtained with the ArmeoSpring for upper-limb assessment after stroke: a reliability and learning effect study for guiding parameter use
Source: J Neuroeng Rehabil. 2020 Sep 29;17:130. doi: 10.1186/s12984-020-00759-2 (PMC7523068; doi:10.1186/s12984-020-00759-2)

# Additional methodological materials

**Kinematic parameters obtained with the ArmeoSpring for upper-limb assessment after stroke: a reliability and learning effect study for guiding parameter use**

Nabila Brihmat[a], Isabelle Loubinoux[a], Evelyne Castel-Lacanal[a,b], Philippe Marque[a,b], David Gasq[a,c]

[a] *ToNIC, Toulouse NeuroImaging Center, University of Toulouse, Inserm, UPS, France.*
[b] *University Hospital of Toulouse, Department of Physical and Rehabilitation Medicine, Toulouse, France.*
[c] *University Hospital of Toulouse, Department of Physiological Explorations, Toulouse, France*

**Additional material S1.** Custom Matlab code used for the calculation of the kinematic parameters, available on GitHub website.

This Matlab script named *Armeo_2DHorizCatch* allows managing the raw data contained in the .csv files generated by the *Armeocontrol 1.22* software provided with the ArmeoSpring.

The raw data are obtained after the completion of the 2D-horizontal catch exercise. One csv file is recorded per trial. The code was designed to import and analyze the data of individual exercises (trials) as part of an assessment session.

The dataset offered with the script, available in the 'Data' folder, allows browsing the csv file content and testing the script.

During the script process, you will be asked to select the csv files and whether you want to visualize graphically the 2D movement trajectories used for the calculation of kinematic parameters and save the plots of these 2D trajectories (in the 'export' folder).

A summary text file containing the computed and averaged parameters for each trial is generated, and saved in the 'export' sub-folder, itself contained in the same folder as the script. If the 'export' folder does not exist, it will be generated automatically.

The script and dataset are freely downloadable on GitHub: https://github.com/davidgasq/Armeo_2DHorizCatch.git

**Additional figure S2.** Box plots comparison between kinematics calculation methods.

Comparison between the averaged parameter values provided in the summary report of the ArmeoSpring (Armeocontrol Software) and those calculated with the Matlab code (custom code). Statistical results of the paired t-tests for the hand path ratio (A. HPR), Task Time (B. TaskTime in seconds) and the Score (C. Score in percentage) are reported on the figure.

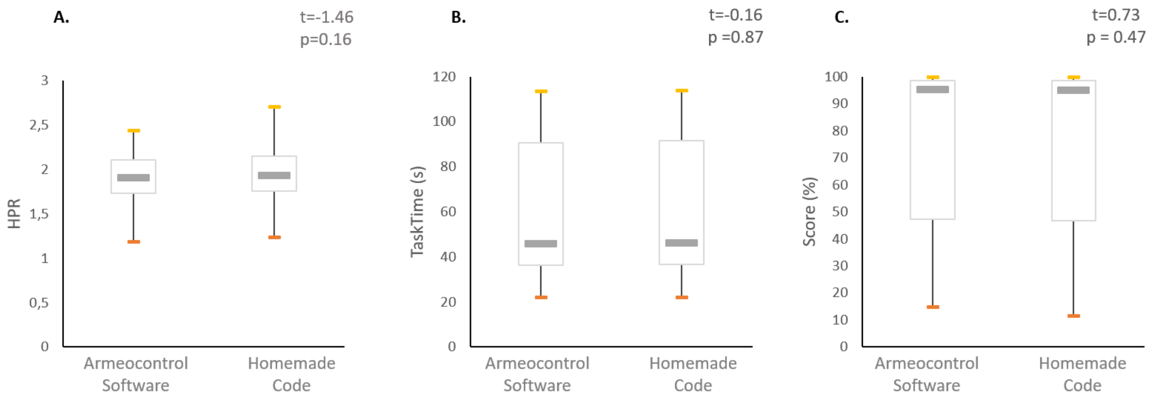


**Additional figure S3.**

Graphical representation of the average number of failed attempts to catch the consecutive balls (targets 1 to 12). Statistical parameters of the ANOVA are shown below the graph. The number of failed attempts to catch the target 1 is significantly lower compared to the other targets (*p<0.0001).

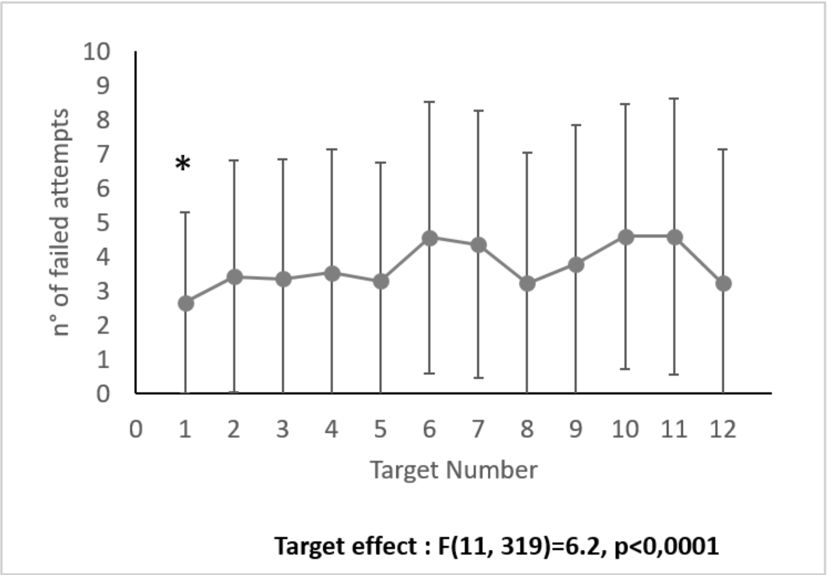

Supplement: Supplementary file 1 — Additional file 1. Additional methodological file. Additional material S1. Custom Matlab code. The Matlab code used for the calculation of kinematic parameters, available on GitHub website. Figure S2. Box plot comparison between kinematics calculation methods. Comparison between the averaged parameter values provided in the summary report of the ArmeoSpring (Armeocontrol Software) and those calculated with the Matlab code (custom code). Statistical results of the paired t-tests for the hand path ratio (A. HPR), Task Time (B. TaskTime in seconds) and the Score (C. Score in percentage) are reported on the figure. Figure S3. Graphical representation of the average number of failed attempts to catch the consecutive balls (targets 1 to 12). Statistical parameters of the ANOVA are shown below the graph. The number of failed attempts to catch target 1 is significantly lower compared to the other targets (*p < 0.0001). [file 12984_2020_759_MOESM1_ESM.pdf]
