# Supplementary material for: Kinematic parameters obtained with the ArmeoSpring for upper-limb assessment after stroke: a reliability and learning effect study for guiding parameter use
Source: J Neuroeng Rehabil. 2020 Sep 29;17:130. doi: 10.1186/s12984-020-00759-2 (PMC7523068; doi:10.1186/s12984-020-00759-2)

Additional results

Kinematic parameters obtained with the ArmeoSpring for upper-limb assessment after stroke: a reliability and learning effect study for guiding parameter use

Nabila Brihmat[a], Isabelle Loubinoux[a], Evelyne Castel-Lacanal[a,b], Philippe Marque[a,b], David Gasq[a,c]

[a] *ToNIC, Toulouse NeuroImaging Center, University of Toulouse, Inserm, UPS, France.*
[b] *University Hospital of Toulouse, Department of Physical and Rehabilitation Medicine, Toulouse, France.*
[c] *University Hospital of Toulouse, Department of Physiological Explorations, Toulouse, France*

Additional figure S4.
Learning curves of two example patients' data (#21 and #29) showing the evolution of a specific parameter over the trials (1 to 10) for the 3 sessions. **A.** Task time (TaskTime, s). **B.** Movement time (MovementTime, s). **C.** Peak velocity (PeakVel, cm/s). **D.** Hand path ratio (HPR, dimensionless). **E.** Number of peak velocity (nPeak). **F.** Game score (Score, %).

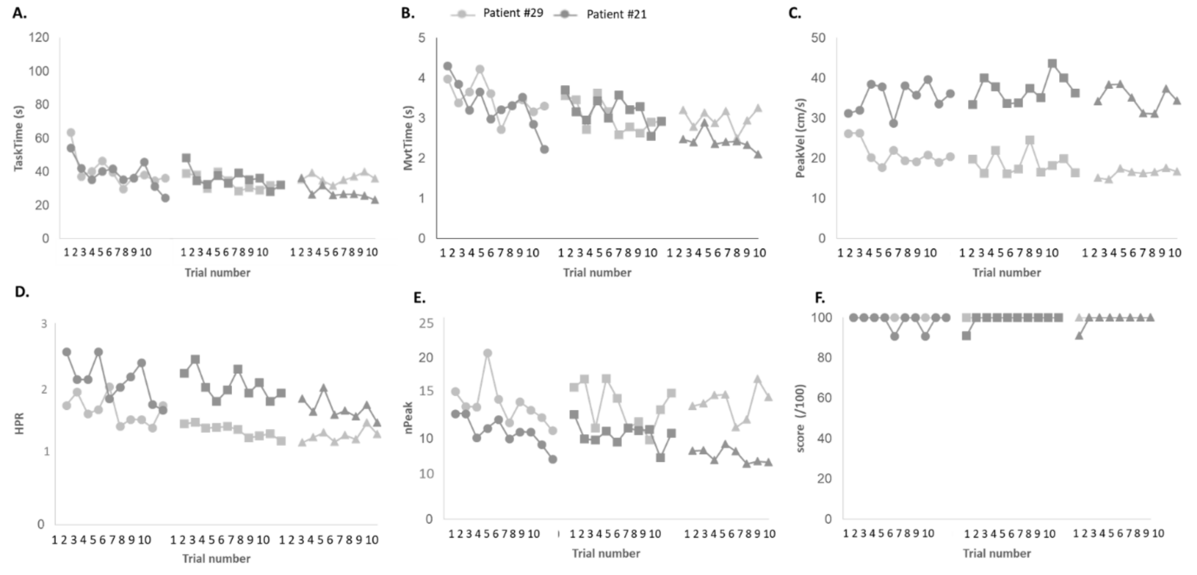

Additional figure S5.

Evolutions of Minimal Detectable Change (MDC, dashed lines) and Intraclass Correlation Coefficient (ICC, solid lines) according to the number of trials (of sessions 2 and 3), considering the calculation of reliability (1: only the 10th trial, to 10: the 10th to the 1st trial). Different parameters are represented by different colors and shapes (black (■): peak velocity, grey (♦): score, orange (●): number of peaks, blue (▲): task time, green (-): movement time, yellow (▬): HPR).

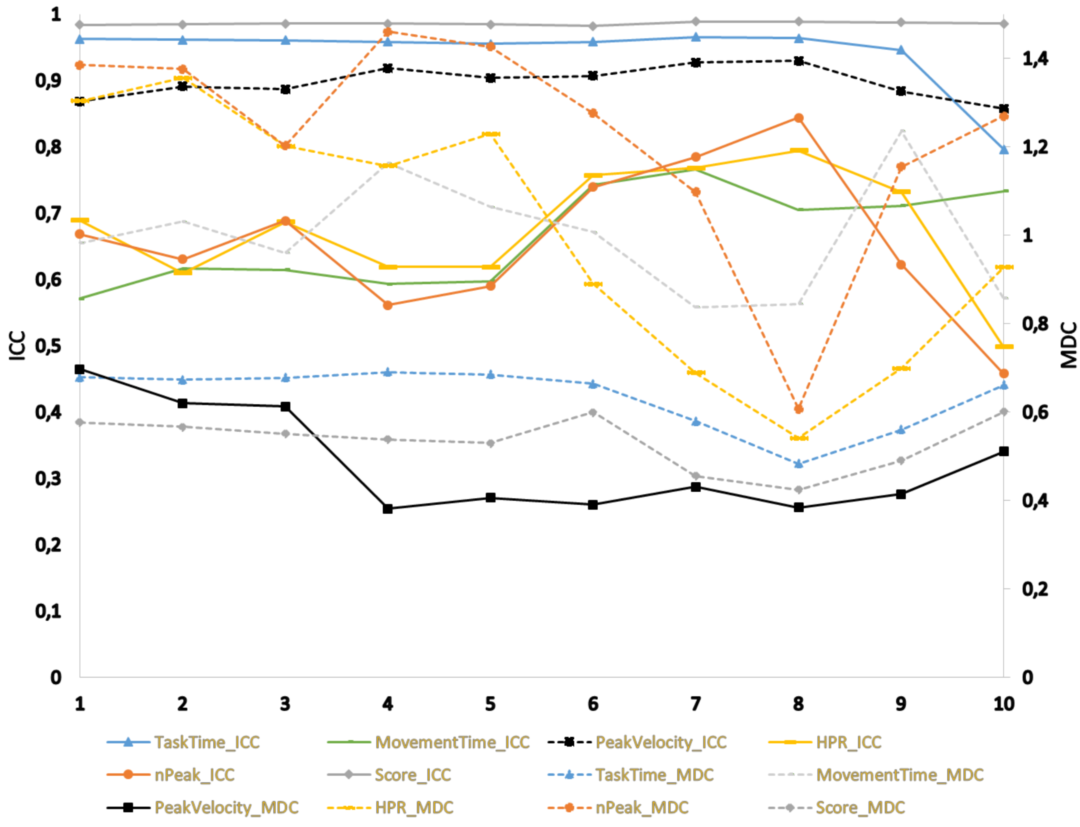

Additional figure S6.

Learning curves of the averaged time of occurrence of the peak velocity during the movement expressed in percentage (*PercPeakVel* in % ±SD), showing the evolution of this parameter over the trials (1 to 10) of the 3 sessions. Between-session significance is represented with asterisks (* $p < 0.05$). No significant trial effect was observed.

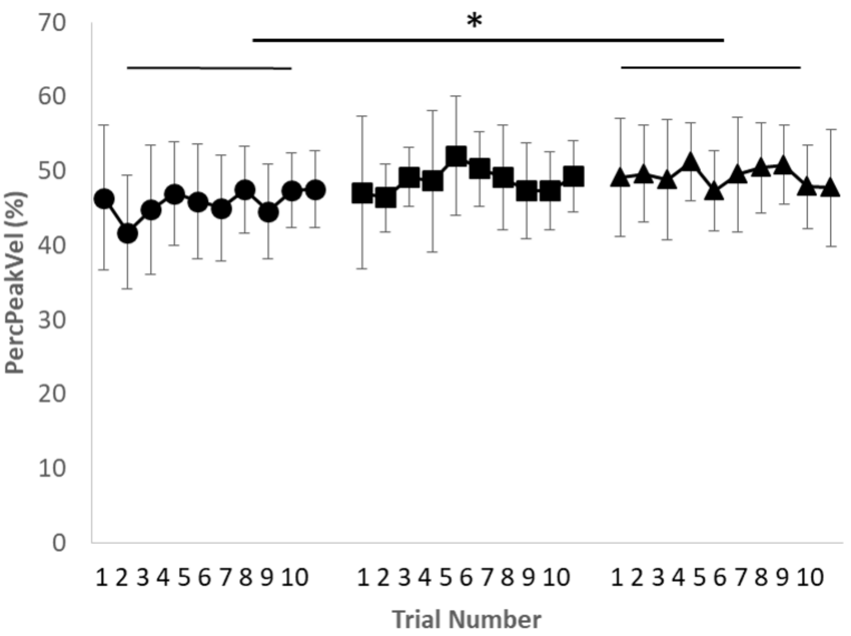

Supplement: Supplementary file 2 — Additional file 2. Additional results file. Figure S4. Learning curves of two example patients’ data (#21 and #29) showing the evolution of a specific parameter over the trials (1 to 10) for the 3 sessions. A. Task time (TaskTime, s). B. Movement time (MovementTime, s). C. Peak velocity (PeakVel, cm/s). D. Hand path ratio (HPR, dimensionless). E. Number of peak velocity (nPeak). F. Game score (Score, %). Figure S5. Evolutions of Minimal Detectable Change (MDC, dashed lines) and Intraclass Correlation Coefficient (ICC, solid lines) according to the number of trials (of sessions 2 and 3), taking into account the calculation of reliability (1: only the 10th trial, to 10: the 10th to the 1st trial). Different parameters are represented by different colors and shapes (black (■): peak velocity, gray (♦): score, orange (●): number of peaks, blue (▲): task time, green (⁃): movement time, yellow (▬): HPR). Figure S6. Learning curves of the averaged time of occurrence of the peak velocity during the movement expressed in percentage (PercPeakVel (%) ±SD), showing the evolution of this parameter over the trials (1 to 10) of the 3 sessions. Between-session significance is represented with asterisks (* p < 0.05). No significant trial effect was observed. [file 12984_2020_759_MOESM2_ESM.pdf]
